# Bacteriological Assessment of the Indoor Air of Different Hospitals of Kathmandu District

**DOI:** 10.1155/2019/5320807

**Published:** 2019-04-08

**Authors:** Arzu Kunwar, Samyukta Tamrakar, Shyaron Poudel, Sony Sharma, Pramila Parajuli

**Affiliations:** Department of Microbiology, St. Xavier's College, Kathmandu 44600, Nepal

## Abstract

Nosocomial infection is the infection that has been caught in a hospital and is potentially caused by organisms that are not susceptible to antibiotics. Nosocomial infections are transmitted directly or indirectly through air and may cause different types of infections. This study was undertaken with an objective to determine the prevalence of nosocomial bacteria present in hospital indoor environment. A total of 16 air samples were taken from general wards and emergency wards of 8 different hospitals using an impactor air sampler in nutrient agar, mannitol salt agar, blood agar, cetrimide agar, and MacConkey agar. The bacteriological agents were isolated and identified by cultural characteristics, Gram staining, and biochemical tests, and their antibiotic susceptibility pattern was determined using CLSI Guideline, 2015. According to the European Union Guidelines to Good Manufacturing Practices, the hospitals were under C- and D-grade air quality. According to the European Commission, most of the hospitals were intermediately polluted. Out of 16 indoor air samples, 47.18% of *Staphylococcus aureus* and 1.82% *Pseudomonas* spp. were isolated. Co*NS*, *Streptococcus* spp., *Micrococcus* spp., and *Bacillus* spp. and Gram-negative bacteria *E.coli* and *Proteus* spp. were identified. The bacterial load was found to be high in the emergency ward (55.8%) in comparison to that in the general ward (44.2%). There is statistically no significant difference between bacterial load and 2 wards (general and emergency) of different hospitals and among different hospitals. The most effective antibiotic against S*. aureus* was gentamicin (81.81%) and ofloxacin (81.81%). Among the antibiotics used for *Pseudomonas* spp., ceftriaxone (83.3%) and ofloxacin (83.3%) were effective. High prevalence of *S. aureus* and Gram-negative bacteria was found in this study; it is therefore important to monitor air quality regularly at different hospitals to prevent HAI.

## 1. Introduction

A hospital-acquired infection (HAI), also known as a nosocomial infection, is an infection that is acquired in a hospital or other healthcare facility. Such an infection can be acquired by susceptible patients in hospital, nursing home, rehabilitation facility, outpatient clinic, or other clinical settings by various means. Healthcare staff can spread infection, in addition to contaminated equipment, bed linens, or air droplets. The infection can originate from the outside environment, another infected patient, staff that may be infected, or in some cases, the source of the infection cannot be determined. In some cases, the microorganism originates from the patient's own skin microbiota, becoming opportunistic after surgery or other procedures that compromise the protective skin barrier. Although the patient may have contracted the infection from their own skin, the infection is still considered nosocomial since it develops in the healthcare setting [1]. The main source of microorganism is human beings as they are discharged through human activities like coughing, sneezing, laughing, and even talking [2].

Nosocomial infections are one of the most serious complications in hospital settings affecting patients in ICU, immunosuppressed people, hospital staff, and people having frequent encounter with healthcare facilities. Nosocomial infections in ICU patients lead to use of broad-spectrum antibiotics and emergence of antibiotic-resistant microorganisms, which ultimately cause high morbidity, mortality, and treatment cost of infection along with a prolonged hospital stay. It has been observed that most prevalent nosocomial infection-causing bacteria like *Staphylococcus aureus* and *Pseudomonas* spp. are developing high multidrug resistance, leading to birth of MDRSA and MDRPA, eventually causing ineffective drug treatment [3].

Gas, dust particles, water vapor, and air contain microorganisms. There are vegetative cells and spores of bacteria, fungi, algae, viruses, and protozoal cysts. Since air is often exposed to sunlight, it has higher temperature and less moisture. Air serves as transport or dispersal medium for microorganism; therefore, they occur in relatively small number in air when compared with soil and water [4].

The air found inside the building is referred to as indoor air. The most common genera of bacteria found in indoor air are *Staphylococci*, *Bacilli*, and *Clostridium* [5]. MRSA (methicillin-resistant *Staphylococcus aureus*) and gentamicin-resistant Gram-negative bacteria are found to be serious nowadays [1]. People spend 80%–90% of their time in indoor environments by breathing on average 14 m^3^ of air per day. Moreover, the environmental and physical factors mainly include temperature, humidity, air exchange rate, air movement, and building structures, location, poor design, ventilation system as well as interior redesign, respectively, which enhance microorganism's growth and multiplication in the indoor atmosphere [5].

A review made by the WHO on the number of epidemiological studies showed that, there is sufficient evidence for an association between indoor dampness-related factors and a wide range of effects on respiratory health, including asthma development, asthma exacerbation, current asthma, respiratory infections, upper respiratory tract symptoms, cough, wheeze, and dyspnoea [6].

Hence, this study provides clear data of microbial air quality and respective bacterial loads in indoor air of hospitals of Kathmandu district. Kathmandu, having an area of 395 km^2^, is the most densely populated district of Nepal. Therefore, we conducted research in hospitals located in comparatively high population density area which represented major areas of Kathmandu district. Identification succeeded by antimicrobial susceptibility pattern of the bacteria isolated from the indoor air of hospitals was determined by using multiple drugs. Globally, the emergence of drug-resistant bacteria is posing as a threat in deliverance of effective medical care. Once a person contracts nosocomial infection, the initial step for management of the infection is antibiotic administration. The commonly used antibiotics for nosocomial agents, *Staphylococcus* spp. and *Pseudomonas* spp., were identified and the susceptibility of the organisms to antibiotics was recorded. Therefore, we studied antibiotic resistance, which is well pronounced in developing countries like Nepal, so as to alert clinicians and assist them in proper treatment decisions and proper management of such patients.

## 2. Materials and Methodology

### 2.1. Materials

#### 2.1.1. Equipment

Impactor air sampler, autoclave, hot air oven, incubator, microscope, refrigerator, weighing machine, gas burners inoculating loop, and wires were used in this study.

#### 2.1.2. Microbiological Media

Nutrient broth, triple sugar iron agar, nutrient agar, simmons citrate agar, mannitol salt agar, sulphur indole motility media, MacConkey agar, oxidative/fermentative agar, urease broth, Mueller-Hinton agar, cetrimide agar, methyl red-Voges-Proskauer broth, and blood agar were used in this study.

#### 2.1.3. Chemicals and Reagents

Barritt's reagent, oxidase reagent, Kovac's reagent, crystal violet, Gram iodine, acetone-alcohol, Safranin, blood plasma, normal saline, hydrogen peroxide, nitrate reagent A, and nitrate reagent B were used in this study.

#### 2.1.4. Antibiotic Discs

All the antibiotic discs used for the susceptibility tests were from HiMedia Laboratories Pvt. Limited, Bombay, India. The antibiotics used were as follows: ampicillin, ofloxacin, erythromycin, ceftriaxone, gentamicin, amikacin, chloramphenicol, ceftazidime, and cotrimoxazole.

### 2.2. Miscellaneous

Conical flasks, cotton, distilled water, dropper, forceps, glass slides, cover slips, immersion oil, Lysol, measuring cylinder, Petri dishes, pipettes, spatula, test tubes, and cotton swab.

## 3. Methodology

### 3.1. Study Area

Two wards—emergency ward and general ward—of different hospitals of Kathmandu district were chosen for the comparative study of hospital indoor air microflora using a microbiological air sampler.

### 3.2. Sample Collection

We performed an active impactor sampling method by using “Hi-Air” air sampler. The height of the sampler is 54 cm, which is also the sampling height. As prementioned, we had only one sampler due to which we collected samples one after another. We allocated two different sites in the emergency and general wards of the hospitals. Sampling from both the wards of one hospital was done in a same day, whereas the sampling from different hospitals was performed on different days.

Impactor samplers use a solid or adhesive medium, such as agar gel. Typically, air is drawn into the sampling head by a pump or fan and accelerated through a perforated plate (sieve samplers) [7]. A standard plate of nutrient agar (total count), mannitol salt agar (specifically for *Staphylococcus aureus*), blood agar (specifically for *Streptococcus* species), MacConkey agar (specifically for Gram-negative bacteria), and cetrimide agar (specifically for *Pseudomonas* species) was aseptically prepared and used.

The impeller speed of 2500 rpm–2600 rpm was so adjusted that 100 liters of air was sampled every minute according to the catalogue of the air sampler. We had a total of 5 plates for each ward, and a single agar plate was placed in the air sampler for 5 minutes so that 500 liters of air was sampled. Therefore, the sampling time at one ward was 25 minutes with a preparation time of 15 minutes. Overall, our sampling period was 80 minutes in one hospital. As we performed sampling on two different days a week so, our total sampling period for 8 hospitals was 1 month.

### 3.3. Transportation of the Sample

Immediately after collection of samples, the Petri plates were taken to the Laboratory of Microbiology of St. Xavier's College. These Petri plates were incubated in an inverted position at 37°C for 24 hrs.

### 3.4. Microbiological Examination of the Sample

After incubation, total plate count was done on the basis of growth on NA plates. The colony characteristics were studied from mannitol salt agar, MacConkey agar, blood agar, and cetrimide agar. After this, the colonies were subjected to Gram staining. Then, for Gram-positive organisms, biochemical tests such as catalase, oxidase, and coagulase and OF tests were performed, whereas for Gram-negative organisms, biochemical tests such as IMViC, TSIA, urease, catalse, oxidase, and OF tests were performed.

### 3.5. Antibiotic Sensitivity Test

Among the identified Gram-positive cocci and Gram-negative bacteria, only *Staphylococcus aureus* and *Pseudomonas* spp. were further tested for their AST pattern, respectively. For this, representative colonies were selected and were suspended in nutrient broth, and the suspension was standardized with respect to 0.5 McFarland solutions. The susceptibility of the isolated organisms towards the antibiotics was tested by using Kirbey–Bauer's Method on Mueller-Hinton agar (CLSI 2015) [8].

For *Staphylococcus aureus,* gentamicin, cotrimoxazole, ampicillin, erythromycin, ofloxacin, and chloramphenicol were used. And for *Pseudomonas* spp., amoxicillin, ceftriaxone, cefotaxime, imipenem, and ofloxacin were used.

### 3.6. Statistical Analysis

The data thus generated were analyzed by simple mean value, percentage, and test of significance by using two-way ANOVA to determine significant differences between the bacterial load and different wards and also between the hospitals where the level of significance was 5% for the analysis.

Test statistics: under H_0_, the two ANOVA F-statistics are

(1) $$\begin{array}{c} F_{\text{cal}}=\frac{\text{MSC}}{\text{MSE}},\\ F_{\text{cal}}=\frac{\text{MSR}}{\text{MSE}}, \end{array}$$

where MSC = mean sum of square of variation due to columns, MSR = mean sum of square of variation due to rows, and MSE = mean sum of square of variation due to errors [9].

### 3.7. Limitations

- As we are undergraduate students, our research work was carried out in college laboratory. There were several group projects simultaneously running in the laboratory where we all shared same space, instruments, and resources like Petri plates, tubes, media, and incubator.
- The project being a part of bachelor's curriculum was conducted alongside our academic classes. Our research time was limited to 5 working days with 5 hours per day in the college laboratory.
- From each air sample plates, we required peculiar colonies on nutrient agar to be further processed and subcultured. Our prospected methodology required around 50–60 number of Petri plates for complete processing of a single air sample plate.

The above-mentioned reasons limited our ability to carry out our duplicate samples. We, therefore, decided to focus on original samples rather than the duplicate samples.

## 4. Results

The following formula was used to calculate bacterial load (cfu/m^3^):

(2) $$\begin{array}{c} \mathrm{Pr}=N\left(\frac{1}{N}+\frac{1}{N-1}+\frac{1}{N-2}+\cdots +\frac{1}{N-r+1}\right), \end{array}$$

where Pr = probable statistical total, *r* = number of CFU counted on 90 mm Petri dish, and *N* = total number of holes in the sampling head = 380 holes [10].

The maximum growth of bacteria was observed in emergency wards (55.72%) as compared to general wards (44.2%) of different hospitals. High bacterial load (348 cfu/m^3^) and low bacterial load (58 cfu/m^3^) were found in the air of hospitals H4 and H7, respectively.

Out of 8 hospitals, general wards of 3 hospitals (H1, H7, and H8) and emergency wards of 3 hospitals (H3, H5, and H7) showed a C-grade air quality. And general wards of 5 hospitals (H2, H3, H4, H5, and H6) and emergency wards of 5 hospitals (H1, H2, H4, H6, and H8) were under D-grade air quality.

Among 8 hospitals, general wards of H7 and H8 and emergency wards of H3 and H7 showed a very low degree of bacterial air pollution (Table 1).

Out of 8 hospitals, *Staphylococcus aureus* was isolated from 7 hospitals including both general and emergency wards. The maximum percentage (10.03%) of *S. aureus* was found to be isolated from the general ward of H2, and the least percentage of isolates (1.21%) was found to be also from the general ward of H6. The result is shown in Table 2.

### 4.1. Occurrence of *Pseudomonas* spp. in Different Hospitals

Out of 8 hospitals, *Pseudomonas* spp. was isolated only from 1 hospital, i.e., H1, in its general ward with the no. of 6 colonies (1.82%).

### 4.2. Occurrence of Gram-Positive Bacteria Other than *S. aureus* in Different Hospitals

Out of 8 hospitals, Co*NS* was isolated from 6 hospitals, followed by *Streptococcus* spp. in 3 hospitals, whereas *Micrococcus* spp. was isolated from only 1 hospital. And *Bacillus* spp. was isolated from 7 hospitals.

### 4.3. Occurrence of Gram-Negative Bacteria Other than *Pseudomonas* spp. in Different Hospitals

Out of 8 hospitals, *E.coli* was isolated from 2 hospitals, and *Proteus* spp. was isolated from only 1 hospital.

Among 8 hospitals, 3 hospitals were divided as hospitals in busy area and 5 were divided as hospitals in less busy area*. S. aureus* was isolated from 3 hospitals in busy area and 4 hospitals in less busy area. *Micrococcus* spp. and *Pseudomonas* spp. were isolated only from H4, semiprivate hospital in busy area and H1, government hospital in less busy area, respectively. The result is shown in Table 3 (busy area refers to the area having high annual patients flow; less busy area refers to the area having comparatively low annual patients flow).

**Figure 1:**
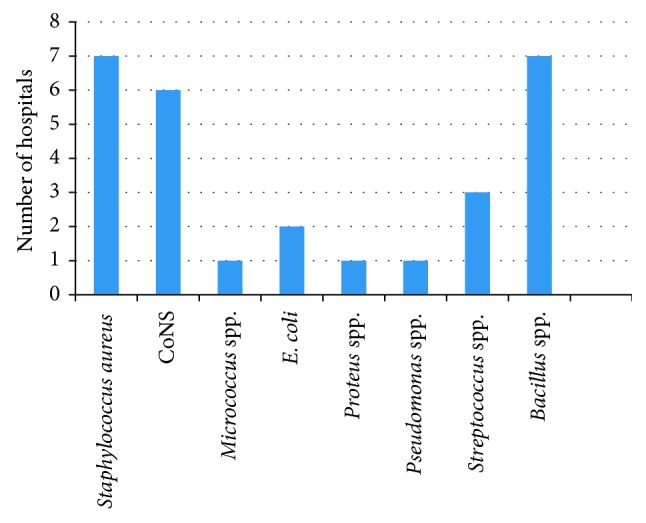
Prevalence of the microorganisms in different hospitals.

Figure 1 demonstrates that the occurrence of *Staphylococcus aureus* was found to be maximum covering 7 out of 8 hospitals, followed by Co*NS* in 6 out of 8 hospitals. *Streptococcus* spp. and *E. coli* were found to be in three and two hospitals, respectively. And the frequency of occurrence of *Micrococcus* spp., *Proteus* spp., and *Pseudomonas* spp. was found to be very least, i.e., only in one hospital.

**Figure 2:**
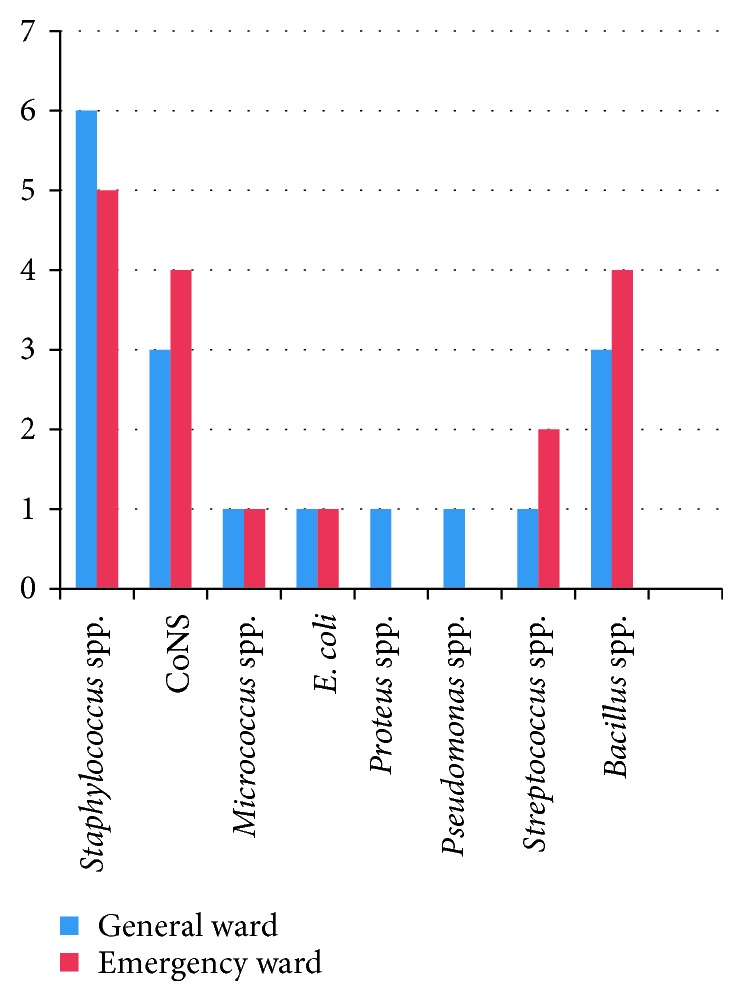
Prevalence of bacteria in general and emergency wards of different hospitals.

Figure 2 demonstrates that *Staphylococcus aureus* was found to be present in general ward and emergency ward of 6 and 5 hospitals, respectively, which was followed by CO*NS* in the general and emergency wards of 3 and 4 hospitals, respectively. However, *Micrococcus* spp. and *E. Coli* were present in both the general and emergency wards of one hospital. In contrast, *Proteus* spp. and *Pseudomonas* spp. were only present in general wards of only one hospital. *Streptococcus* spp. was present in general and emergency ward of 1 and 2 hospitals. And *Bacillus* spp. was present in both the general and emergency ward of 3 and 4 hospitals, respectively.

The most effective antibiotic against isolated *Staphylococcus aureus* was gentamicin (81.81%) and ofloxacin (81.81%) and the least effective was chloramphenicol (36.36%) and erythromycin (36.36%).

The most effective antibiotic against isolated *Pseudomonas* spp. was ceftriaxone (83.3%) and ofloxacin (83.3%) and least effective was cefotaxime (16.6%).

## 5. Discussion

Microbiological quality assessment of indoor air is one of the most vital investigations to determine the microbial indoor air pollution. The information on the indoor microbial concentrations of airborne bacteria is necessary both to estimate the health hazard and to create standards for indoor air-quality control [5].

There was a presence of certain bacterial load in emergency and general wards of 8 hospitals where the study was carried out. From Table 4, we can observe that among the two wards, emergency ward was found to have high bacterial load (55.72%) in comparison to general ward (44.2%). A contrast result was obtained in a study conducted by Awosika et al. [11], where out of nine wards, the high bacterial load was found to be in medical ward (25%), whereas the least bacterial load was recorded in emergency unit (2%). Humid room environment, presence of unhygienic attached toilets, poor waste management system, and high number of patients in a single room, personnel, and visitors occupying the hospital might be the reasons for high bacterial load in emergency ward in this study.

According to the European Union Guidelines to Good Manufacturing Practices (Table 5), indoor air possessing greater than 100 cfu/m^3^ and 200 cfu/m^3^ bacterial loads is referred to as C- and D-grade air quality, respectively. In our study, Table 1 demonstrates that all the general wards and emergency wards of 8 hospitals were under C- and D-grade air quality. This might be because at the time of this study, all wards were at their maximum capacity, as of visitors in and out the wards, the high density of patients in the wards which resulted in more shedding of bacteria and agitation of air. Besides, the environmental factors led to poor indoor air quality.

Among all the 8 hospitals studied, general ward of H7 and H8 and emergency ward of H3 and H7 comparatively showed a good air quality. According to Sanitary Standards for NonIndustrial Premises, European Commission (Table 6), indoor air having bacterial load <50 cfu/m^3^ is considered to be of good air quality. Most of the hospitals as shown in Table 7 were under intermediate degree of air pollution as the bacterial load present in those hospitals was in the range of 100–500 cfu/m^3^. According to the work conducted by WHO experts, total bacterial load should not exceed 1000 cfu/m^3^. Similarly, in this study, all the hospitals had bacterial load less than 1000 cfu/m^3^, indicating a satisfactory level of air quality.

Out of 8 hospitals, which can be seen in Table 2, *Staphylococuus aureus* (47.18%) was isolated from 7 hospitals. Among the two wards, general ward showed a high occurrence of *S. aureus* (28.85%) than emergency ward (18.33%). *S. aureus*, a normal flora of the human body residing in the nasal passage, is a leading pathogen causing nosocomial infection because of which among all the Gram-positive cocci isolated, *S. aureus* was the targeted Gram-positive organism of this study. The most common source of *S. aureus* is sneezing. In a similar study done by Qudiesat et al. [14] in selected hospitals of Zarqa city, Jordan, *S. aureus* (16.2%) was found to be the predominant organism.

Other isolated and identified Gram-positive cocci in this study were Co*NS*, *Streptococcus* spp., and *Micrococcus* spp. Out of 8 hospitals, they were isolated from 6, 3, and 1 hospitals, respectively, interpreted in Table 2. In a study carried out by Sapkota et al. [15], they screened out *S. aureus* (57.1%), followed by *Micrococcus* spp. (26%), Co*NS* (29.9%), and Gram-negative bacteria from the air culture of government hospitals of Nepal. *Streptococcus* spp., a causative agent of severe pneumonia associated with nosocomial infection, was isolated from 3 hospitals. *Bacillus* spp. was also predominantly isolated from 5 hospitals. Common species of *Bacillus* exhibit a wide range of physiologic abilities that allow them to live in every natural environment [8].

In this study, *Pseudomonas* spp. was isolated from general ward of only one hospital H1 (1.82%), given within Table 2. A contrast result was obtained in a study carried out by Nandalal and Somashekar [16] where the prevalence of *Pseudomonas* spp. was found in almost all sites (38 cfu/m^3^). The occurrence of *Pseudomonas* spp. in only H1 in our study might be due to poor sanitation of hospital environment and medical equipment. There was a lack of regular risk assessment of water system of the hospital (H1) building due to which we observed stagnant water in the hospital's toilets and bathrooms. Stagnant water is known as breeding ground for *Pseudomonas* spp. [17] and this could be a cause of persistence of this bacterium in this hospital. Also, presence of patients infected with *Pseudomonas* spp. within the sampling site might have led to occurrence of *Pseudomonas* spp. in our study.

*E. coli* was isolated from both wards of one hospital, and *Proteus* spp. was also isolated from only one hospital as shown in Figure 2. The presence of members of Enterobacteriaceae in this study might be due to the presence of toilets in the same hall of emergency ward which led to the direct contact of the patients with the organisms as these are the intestinal microflora of our human body which eventually led to the airborne condition of these organisms.

As shown in Table 3, this study categorizes the hospitals on the basis of the crowd of people and traffic of vehicles as busy and less busy. We targeted hospitals located in areas having high population density. And then after surveying their annual report on the patients flow, we ranked hospitals on the basis of obtained data ultimately categorizing them into busy and less busy. In our study, the busy hospitals had an annual patient's flow ranging from around 13,000 up to 4, 00,000 and the less busy hospitals had an annual patients flow from around 4,000 up to 7000. Therefore, busy hospitals are those hospitals located in highly crowded location in terms of people and vehicles. Another classification presented in Table 3 of the hospitals was done on the basis of Government, private, and semiprivate organizations. Among the hospitals located in busy area, H5, a private hospital, showed a high prevalence of *S.aurues*, whereas H4 being a semiprivate hospital showed a presence of *Micrococcus* spp. Similarly, among the hospitals located in less busy area, H2, a government hospital, had high prevalence of *S. aureus*, and *Pseudomonas* spp. was isolated only from H1, which is also a government hospital located in less busy area.

The dampness situation of the building might have created favorable condition for the bacterial growth, which can be dispersed through droplets and then maintained in aerial suspension which can have health risk among people. The relative humidity of the air has shown to be of major importance in the survival of microorganisms. The mechanism is totally related to organism's surface biochemistry. One mechanism that explains the loss of viability in association with very low relative humidity is structural change in the lipid bilayer of the cell membrane as the water is lost from the cell. Cell membrane bilayer changes from the typical crystalline structures to the gel phase. This structural phase transition affects cell surface protein configuration and ultimately results in inactivation of the cell. In general, Gram-negative bacteria react unfavorably to the desiccation, whereas Gram-positive bacteria are more tolerant to desiccation [18]. This can be possible reasoning for more prevalence of Gram-positive bacteria. Hence, the most important means for avoiding adverse health effects is minimization of persistent dampness and microbial growth on interior surfaces and in building structures. And also, it was stated by the WHO that dampness situation has to be considered as the risk indicator for health risk of biological contaminants of indoor air [5].

*F* test was applied to study the difference in air quality between the wards of every hospital and between hospitals. By using the formula mentioned in (1), *F*_cal_ and *F*_tab_ were calculated. Since *F*_cal_ (0.92, 0.0131) is less than *F*_tab_ (5.991, 3.79), the null hypothesis H0 is accepted. The statistical analysis showed that there is no significant difference in the concentrations of bacteria between general and emergency wards. This explains that both the wards of all the eight different hospitals, which were studied, had a similar bacterial load statistically. Similarly, there are no significant differences in the concentrations of bacteria between the hospitals.

In this study, gentamicin and ofloxacin were found to be 81.81% effective towards isolates of *S. aureus* as shown in Table 8. Hence, it can be used as drug of choice for the treatment of nosocomial infections caused by *S.aureus*. Isolated *S. aureus* was found to be less susceptible to erythromycin (36.36%) and chloramphenicol (36.36%).

Asia is one of the epicenters of antimicrobial resistance worldwide, and this is an increasing public health concern. MDR pathogens have been widely disseminated, both in hospitals and throughout communities, in many countries [19]. The relative frequency of *P. aeruginosa* as a nosocomial pathogen has increased, although wide variations are seen among individual medical centers. *Pseudomonas aeruginosa* continues to be a major pathogen among patients with immunosuppressant, cystic fibrosis, malignancy, and trauma [20]. Despite the high prevalence of MDR or XDR *Pseudomonas* in Asia, the clinical consequences of antimicrobial resistance are not fully understood in many Asian countries. In a Korean hospital, antimicrobial resistance, especially to ceftazidime and imipenem, adversely affected the outcomes of patients with *Pseudomonas aeruginosa* bacteraemia. Rates of carbapenem-resistant *Pseudomonas* were very high, and MDR nonfermenters were highly prevalent in Asian countries [20]. In this study (Table 9), *Pseudomonas* spp. was highly susceptible to ceftriaxone (83.3%) and ofloxacin (83.3%). And it was resistant to imipenem. It might be because of the mutation of genes. Due to the higher sensitivity towards ceftriaxone and ofloxacin, it can be used as a drug of choice for the treatment of nosocomial infections caused by this bacterium (Tables 10 and 11).

## 6. Conclusion

From the 8 hospital samples processed in this study, 245 isolates of 8 different bacterial species were obtained. Both of the wards (general and emergency) of all the eight hospitals were found to be under C- and D-grade of air quality. According to Sanitary Standard for Non-Industrial Premises, European Commission, the majority of general wards of 5 hospitals were found to be intermediately polluted and similar results were found in the case of emergency wards of 5 hospitals. High bacterial load was found to be present in H4 (348 cfu/m^3^), which is a semiprivate hospital located in busy area. And low bacterial load was found in H7 (58 cfu/m^3^), which is a private hospital located in less busy area. *Staphylococcus aureus* (47.18%) was one of the major organisms isolated from 7 out of 8 hospitals. *Pseudomonas* spp. (1.82%) was isolated from general ward of only one hospital. Out of 8 hospitals, Co*NS* was isolated from 6 hospitals, followed by *Streptococcus* spp. in 3 hospitals, whereas *Micrococcus* spp. was isolated from only 1 hospital and *Bacillus* spp. was isolated from 7 hospitals (Figure 1). Out of 8 hospitals, *E.coli* was isolated from 2 hospitals, and *Proteus* spp. was isolated from only 1 hospital. While performing the antibiotic susceptibility test for *S. aureus*, it was found to be highly susceptible to gentamicin (81.81%) and ofloxacin (81.81%), whereas it was less susceptible to erythromycin (36.36%) and chloramphenicol (36.36%). Similarly, in the case of AST of *Pseudomona*s spp., it was found to be susceptible to ceftriaxone (83.3%) and ofloxacin (83.3%), whereas *Pseudomonas* spp. did not show susceptibility against imipenem. The high bacterial concentrations of air obtained in this study might be potential risk factors for spread of nosocomial infection in respective hospitals.

## Figures and Tables

**Table 1:** Grading of hospitals according to European Union Guidelines to Good Manufacturing Practices.

S. N	Hospitals	Number of colonies in general ward (calculated)	Air quality (grade)	Number of colonies in emergency ward (calculated)	Air quality (grade)
1	H1	96	C	116	D
2	H2	172	D	128	D
3	H3	132	D	26	C
4	H4	206	D	142	D
5	H5	110	D	82	C
6	H6	108	D	114	D
7	H7	28	C	30	C
8	H8	44	C	102	D
*Total*		**896**		**740**	

**Table 2:** Number and percentage of *Staphylococcus aureus* in different wards of the hospital.

S. N	Hospitals	Number of organism (%)	
		Emergency ward	General ward
1	H1	Nil	19 (5.77)
2	H2	20 (4.83)	33 (10.03)
3	H3	Nil	Nil
4	H4	18 (4.34)	11 (3.34)
5	H5	16 (3.86)	23 (6.99)
6	H6	16 (3.86)	4 (1.21)
7	H7	6 (1.44)	Nil
8	H8	Nil	5 (1.51)
*Total*		**76**	**95**

The percentage of isolated *S. aureus* was calculated on the basis of total plate count on NA.

**Table 3:** Prevalence of *S. aureus* and *Pseudomonas* spp. in context with the condition and type of the organization.

S. N	Condition	Organization	Hospitals	Number of organisms	
				*S. aureus*	*Pseudomonas* spp.
1	Busy	Government	—	—	—
		Private	H5	39	Nil
			H6	20	Nil
		Semiprivate	H4	29	Nil

2	Less busy	Government	H1	19	6
			H2	53	Nil
		Private	H7	6	Nil
			H8	5	Nil
		Semiprivate	H3	Nil	Nil

**Table 4:** Distribution of bacterial load in different wards of different hospitals.

S. N	Hospitals	Number of colonies (observed)		Number of colonies (calculated) (cfu/m^3^)		Total
		Emergency ward	General ward	Emergency ward	General ward	
1	H1	43	52	96	116	212
2	H2	75	57	172	128	300
3	H3	59	11	132	26	158
4	H4	88	63	206	142	348
5	H5	49	37	110	82	192
6	H6	48	51	108	114	222
7	H7	12	12	28	30	58
8	H8	42	46	44	102	146
*Total*		**414 (55.72%)**	**329 (44.2%)**			

**Table 5:** Standards for air quality evaluation according to European Union Guidelines to Good Manufacturing Practice (European Commission, 2008) [12].

S. N	Grade	Cfu/m^3^	Cfu/plate
1	A	<1	<1
2	B	10	5
3	C	100	50
4	D	200	100

**Table 6:** Evaluation of air quality according to the sanitary standards for nonindustrial premises (CEC, 1993) [13].

S. N	Group of microbes	Range values (Cfu/m^3^)	Air pollution degree
1	Bacteria	<50	Very low
2	Bacteria	50–100	Low
3	Bacteria	100–500	Intermediate
4	Bacteria	500–2000	High
5	Bacteria	>2000	Very high

**Table 7:** Air pollution degree according to Sanitary Standards For Non-Industrial Premises, European Commission.

S. N	Hospitals	Number of colonies in general ward (calculated)	Air pollution (degree)	Number of colonies in emergency ward (calculated)	Air pollution (degree)
1	H1	96	Low	116	Intermediate
2	H2	172	Intermediate	128	Intermediate
3	H3	132	Intermediate	26	Very low
4	H4	206	Intermediate	142	Intermediate
5	H5	110	Intermediate	82	Low
6	H6	108	Intermediate	114	Intermediate
7	H7	28	Very low	30	Very low
8	H8	44	Very low	102	Intermediate
*Total*		**896**		**740**	

**Table 8:** Antibiotic susceptibility pattern of *Staphylococcus aureu*s.

Antibiotics used	Sensitive		Intermediate		Resistant		Total
	Number	(%)	Number	(%)	Number	(%)	
Gentamicin	9	81.81	1	6.25	1	6.25	11
Cotrimoxazole	5	45.45	2	18.18	4	36.36	11
Ampicillin	6	54.54	3	27.27	2	18.18	11
Erythromycin	4	36.36	5	45.45	2	18.18	11
Ofloxacin	9	81.81	0	0	2	18.18	11
Chloramphenicol	4	36.36	3	27.27	4	36.36	11

**Table 9:** Antibiotic susceptibility pattern of *Pseudomonas* spp.

Antibiotics	Sensitive		Intermediate		Resistant		Total
	Number	(%)	Number	(%)	Number	(%)	
Amoxycillin	4	66.67	2	33.3	0	0	6
Ceftriaxone	5	83.3	1	16.6	0	0	6
Cefotaxime	1	16.6	2	33.3	3	50	6
Imipenem	0	0	0	0	6	100	6
Ofloxacin	5	83.3	1	16.6	0	0	6

**Table 10:** Zone of size standard interpretative chart (HiMedia Catalogue 2017-18) *Staphylococcus* spp. [21].

Antimicrobial agent	Symbol	Disc content	Resistant (mm or less)	Intermediate (mm)	Sensitive (mm or more)
Ampicillin	AMP	10 *μ*g	28	—	29
Chloramphenicol	C	30 *μ*g	12	13–17	18
Cotrimoxazole	COT	25 *μ*g	10	11–15	16
Erythromycin	E	15 *μ*g	13	14–22	23
Gentamicin	GEN	10 *μ*g	12	13–14	15
Ofloxacin	OF	5 *μ*g	14	15–17	18

**Table 11:** Zone of size standard interpretative chart (HiMedia Catalogue 2017-18) *Pseudomonas* spp. [21].

Antibiotics	Sensitive	Intermediate	Resistant
Amoxycillin	17	14–16	13
Ceftriaxone	21	14–20	13
Cefotaxime	23	15–22	14
Imipenem	16	14-15	13
Ofloxacin	16	13–15	12

## Data Availability

Further data related to this study can be made available upon request.

## References

[1] Srikanth P., Sudharsanam S., andSteinberg R. (2008). Bio-aerosols in indoor environment: composition, health effects and analysis. *Indian Journal of Medical Microbiology*.

[2] Klevens R. M., Edwards J. R., Richards C. L (2007). Estimating health care-associated infections and deaths in U.S. hospitals. *Public Health Reports*.

[3] Ozer B., Akkurt B. C. O., Duran N., Onlen Y., Savas L., Turhanoglu S. (2011). Evaluation of nosocomial infections and risk factors in critically ill patients. *Medical Science Monitor*.

[4] (2015). *Isolation and Identification of Air Microflora in Microbiology Laboratoryosun State Polytechnic, researchClue.com, Iree, Nigeria*.

[5] Hayleeyesus S. F., Manaye A. M. (2014). Microbiological quality of indoor air in university libraries. *Asian Pacific Journal of Tropical Biomedicine*.

[6] Mendell M. J., Mirer A. G., Cheung K. (2009). Health effects associated with dampness and mould. *Who Guidelines for Indoor Air Quality: Dampness and Mould*.

[7] Lawley R. (2009). Something in the air: monitoring airborne microorganisms. *Food Engineering and Ingredients*.

[8] Clinical and Laboratory Standards Institute (CLSI) Antimicrobial Susceptibility Testing Standards, 2015

[9] Sthapit A. B., Yadav R. P., Khanal S. P., Dongol P. M. (2014). *Applied Statistics: Analysis of Variance-ANOVA*.

[10] Anderson A. A. (1958). New sampler for the collection, sizing, and enumeration of viable air borne particles. *Journal of Bacteriology*.

[11] Awosika S. A., Olajubu F. A., Amusa N. A. (2012). Microbiological assessment of indoor air of a teaching hospital in Nigeria. *Asian Pacific Journal of Tropical Biomedicine*.

[12] European Commission Enterprise and Industry Directorate (2008). *General Volume 4 EU Guidelines to Good Manufacturing Practice*.

[13] CEC, Commission of the European Communities (1993). Indoor air quality and its impact on man.

[14] Qudiesat K., Abu-Elteen K., Elkarmi A., Hamad M., Abusasaud M. (2009). Assessment of airborne pathogens in healthcare settings. *African Journal of Microbiology Research*.

[15] Sapkota B., Gupta G. K., Shrestha S. K., Pradhan A., Karki P., Thapa A. (2016). Microbiological burden in air culture at various units of a tertiary care government hospital in Nepal. *Australasian Medical Journal*.

[16] Nandalal P., Somashekhar R. K. (2007). Prevalence of Staphylococcus aureus and Pseudomonas aeruginosa in indoor air flora of a district hospital, Mandya, Karnataka. *Journal Of Environmental Biology*.

[17] Special Report: Pseudomonas in Hospitals, https://www.buildingbetterhealthcare.co.uk/news/article_page/Special_report_Pseudomonas_in_hospitals/75712

[18] Pepper I. L., Dowd S. E. (2009). *Aero Microbiology-Environmental Microbiology*.

[19] Cross A., Allen J. R., Burke J. (1983). Nosocomial infections due to pseudomonas aeruginosa: review of recent trends. *Clinical Infectious Diseases*.

[20] Kang C. I., Song J. H. (2013). Antimicrobial resistance in Asia: current epidemiological and clinical implications. *Infection & Chemotherapy*.

[21] HiMedia *Catalogue Antimicrobial Testing–Zone Size Interpretative Chart 237–294*.

